# Mapping the pathway and support offered to children with an intellectual disability referred to specialist mental health services in the UK

**DOI:** 10.1192/bjb.2024.63

**Published:** 2025-06

**Authors:** Vaso Totsika, Zhixing Yang, Lauren Turner, Charmaine Kohn, Angela Hassiotis, Eilis Kennedy, Michael Absoud, Rachel McNamara, Elizabeth Randell, Sophie Levitt, Gemma Grant, Angela Casbard, Lauris Jacobs, Cristina Di Santo, Claire Buckley, Emma Hignett, Ashley Liew

**Affiliations:** 1 University College London, UK; 2 Tavistock & Portman NHS Foundation Trust, London, UK; 3 South London and Maudsley NHS Foundation Trust, London, UK; 4 Cardiff University, UK; 5 Brighter Futures for Children, Reading, UK; 6 Challenging Behaviour Foundation, Chatham, UK; 7 Alder Hey Children's NHS Foundation Trust, Liverpool, UK; 8 Lancashire & South Cumbria NHS Foundation Trust, Preston, UK

**Keywords:** CAMHS, intellectual disability, behaviour that challenges, mental health

## Abstract

**Aims and method:**

This survey of 66 specialist mental health services aimed to provide an up-to-date description of pathways of care and interventions available to children with an intellectual disability referred for behaviours that challenge or with suspected mental health problems.

**Results:**

Overall, 24% of services made contact with a family at referral stage, whereas 29% contacted families at least once during the waiting list phase. Only two in ten services offered any therapeutic input during the referral or waiting list stages. During the active caseload phase, services offered mostly psychoeducation (52–59%), followed by applied behaviour analytic approaches for behaviours that challenge (52%) and cognitive–behavioural therapy (41%). Thirty-six per cent of services had not offered any packaged or named intervention in the past 12 months.

**Clinical implications:**

With increasing waiting times for specialist mental health support, services need to consider increasing the amount of contact and therapeutic input on offer throughout all stages of a child's journey with the service.

Children with an intellectual disability (learning disability in the UK) who experience mental health problems or present with behaviours that challenge are typically referred to specialist mental health services for assessment and support. However, evidence indicates that only about 30% of children with intellectual disability and mental health problems access specialist mental health support,^1^ whereas fewer than 20% of families access a named or packaged intervention.^2^

Service provision within specialist mental health services is not uniform. There is wide variation within pathways of care, including what happens following a referral for suspected mental health problems or behaviours that challenge and what therapeutic interventions children and families may expect to be offered following assessment. Variation in service pathways may, in part, be related to policy and practice initiatives.^3,4^ Available information on pathways of care is scant but much needed by researchers, clinicians and commissioners to improve care for that group of children and their parents.

The aim of the present study was to provide an up-to-date description of service pathways and the support offered to families whose child with an intellectual disability has been referred to a specialist mental health service. The study captured information on the amount of contact services have with families in the early stages of a referral, as well as information on support and interventions offered at all stages of a child's journey with the service (referral stage, waiting list and active case load). Findings from the present study identify service gaps and could be used to improve local offers and provide directions for future research.

## Method

### Participants and settings

A total of 93 professionals participated in the survey. Most were child and adolescent psychiatrists (*n* = 20) or clinical or educational psychologists (*n* = 23). As the survey was anonymous, no personal information about participants was collected. Services were located across the UK, with seven services in Scotland, five in Northern Ireland, two in Wales and 68 in England. In terms of the type of service, 34 participants (37%) indicated that their service was a learning disability child and adolescent mental health service (CAMHS), 11 (12%) came from a CAMHS with a pathway for neurodevelopmental disorders and learning disability, and one (1%) came from a CAMHS with a neurodevelopmental disorders pathway. The service types selected from the options offered in the survey were: neurodevelopmental paediatrics (*n* = 8, 9%), neurodevelopmental paediatrics with integrated mental health pathway (*n* = 2, 2%), community learning disability team (with separate child pathway; *n* = 7, 8%) and positive behaviour support team (all ages with a separate child pathway, *n* = 4, 4%). About 14% selected ‘other’ (*n* = 14) and provided the name of their service – these free-text data were later coded; see ‘Approach to analysis’. Responses regarding type of service were missing for 12 participants (13%).

### Measures

#### Assessment of intellectual disability

Participants were asked to indicate ways in which they make a diagnostic assessment of intellectual disability during routine care within their service. Responses included ‘Adaptive behaviour measure and cognitive assessment’, ‘Adaptive behaviour measure and informal observation’, ‘Cognitive assessment and history on adaptive functioning’, ‘School information history and informal observation’ and ‘My service does not assess for intellectual disability’.

#### Amount of contact with a family

Participants were asked to indicate the amount of contact the service has with a family whose child has been referred to the service or is on the waiting list for further assessment or to begin treatment. Participants could select: ‘No contact from our service’, ‘To be contacted 1–2 times’, ‘To be contacted over 2 times’, ‘It varies, I cannot say’ and ‘Our service does not have a referral/waiting list’.

#### Intervention and support offered

Participants indicated the types of intervention approaches and support offered during the three stages of their contact with the service: at referral, waiting list and active caseload. For each stage, participants were asked whether their service offers any therapeutic or psychoeducation support (yes/no). If participants indicated ‘yes’, they were presented with a structured list of 22 approaches (e.g. psychoeducation, the family gets a leaflet or a phone call on what the service offers) or specific and/or named interventions (e.g. Stepping Stones Triple P) and asked to select any their service offered in the past 12 months. The structured list of approaches and interventions was initially developed based on findings from a previous study^2^ but was then adapted and refined following piloting with three clinical services.

Participants provided any other information they wanted in relation to contact with families or intervention offered to a referred case. Free-text data were coded to identify any specific intervention that had not been included in the structured list. Drawing on free-text data and responses to the structured list mentioned above, we identified whether any intervention was made available to a family at each stage of the contact with a service (referral list, waiting list and active caseload list). Intervention was defined as a named approach (e.g. child psychotherapy) or a manualised and/or packaged intervention (e.g. cognitive–behavioural therapy), in contrast to signposting only (e.g. multi-agency liaison), non-packaged and/or non-manualised psychoeducational input (e.g. psychoeducation: the family gets a leaflet or a phone call on what the service offers) or no intervention offered. Coding of the variable ‘any intervention offered’ across the free-text and structured list data was undertaken by the research group as a whole (not individual researchers).

#### Patient and participant involvement

A group of ten parent carer advisors contributed to analysis, interpretation and write-up of study findings. The focus on specialist mental health services separately from services without a distinct mental health pathway was suggested by this group.

### Procedure

Data were collected through a survey between November 2022 and January 2023. The survey invited staff working in UK specialist mental health services for children with an intellectual disability to take part. Staff were invited to participate if they worked in a service that accepted referrals for children with an intellectual disability and challenging behaviour or suspected mental health problems. Participation was anonymous. Potential participants were told they would be asked about their role and service type and information on service input during the course from referral to waiting list to active case load. The survey was hosted by REDCap, a secure web application, and it was advertised through social media, newsletters and mailing lists of professional organisations (e.g. Child and Adolescent Intellectual Disability Psychiatry Network, British Association for Community Child Health; CAMHS Network). The recruitment approach meant that it was not possible to estimate the number of potential participants who may have seen the survey call.

### Approach to analysis

Descriptive statistics were used to describe (a) diagnostic practices; (b) amount of contact between families and services at referral and waiting list stage and support offered during this stage; and (c) interventions offered at each of the three stages of a family's journey with a service, i.e. referral, waiting list and active caseload.

Data on service type ‘other’ were coded to distinguish between specialist mental health services and those services that did not have a distinct mental health or challenging behaviour pathway for children with learning disability (e.g. children's community therapies teams, speech and language service). A total of 66 respondents were identified as coming from a specialist mental health service. The coding of service type into specialist mental health service versus not a specialist mental health service was undertaken by the research team as a whole. Table 1 includes a description of participants and settings for the 66 specialist mental health services. Descriptive statistics are accompanied where relevant by verbatim quotes from free-text data to contextualise information provided by participants.

**Table 1:** Participants and settings – specialist mental health services

	*N* = 66	%
Role in service		
Service manager	1	2
Child and adolescent psychiatrist	20	30
Paediatrician	1	2
Clinical or educational psychologist	22	33
Speech and language therapist	7	11
Occupational therapist	3	5
Nurse or learning disability nurse	10	15
Learning disability psychiatrist	1	2
Other allied health professional	1	2
Services		
Learning disability child and adolescent mental health service (CAMHS)	36	55
CAMHS with learning disability/neurodevelopmental pathway	13	20
Child disability team with learning disability/neurodevelopmental expertise (children's learning disability team, neurodevelopmental paediatrics with integrated mental health pathway, paediatric liaison in acute mental health services, tertiary level neurodevelopmental service)	6	9
Community learning disability team; all ages (including pathway for children with learning disabilities)	7	11
Positive behaviour support team; all ages (including pathway for children with learning disabilities)	4	6

### Ethics

The survey reported in this study was conducted anonymously and no personal data were collected. The research did not require review by an ethics committee. The study was conducted as part of a larger project which has been reviewed by the London-South East Research Ethics Committee (IRAS ID 315829; approval 12/12/2022).

## Results

### Diagnostic practices

About one-third (29%) of specialist mental health services reported undertaking a formal assessment to diagnose intellectual disability (a formal assessment is a combination of standardised cognitive assessment and standardised adaptive skills assessment).^5^ A quarter (24%) did not conduct intellectual disability diagnoses.

### Contact with and support for families on referral and waiting lists

Respondents were asked to indicate the amount of contact a family is expected to receive from their service while waiting for the referral to be evaluated (referral list) and after the referral has been accepted and the child is awaiting assessment/input (waiting list). The most frequently selected response was ‘It varies, I cannot say’; this was indicated by approximately 30% of respondents (Table 2). Free-text data from one respondent indicated that ‘contact varies on if further information is needed’. The second most frequent response was no response (missing data), and 14% and 9% of participants indicated that there is no contact at all during referral or waiting list stages, respectively. Between 24% and 29% of services contacted families at least once (Table 3).

**Table 2:** Contact with families when a child is on the referral or waiting list for specialist mental health services (*N* = 66)

	Referral list	Waiting list
No contact	9 (14%)	6 (9%)
Contact 1–2 times	10 (15%)	11 (17%)
Contact more than 2 times	6 (9%)	8 (12%)
Our service does not have a referral/waiting list	10 (15%)	4 (6%)
It varies, I cannot say	22 (33%)	20 (30%)
Missing data	9 (14%)	17 (26%)

**Table 3:** Interventions and supports offered at each stage of a child's contact with a specialist mental health service

	Referral	Waiting list	Active caseload
Psychoeducation (leaflet or phone call on what is offered)	9 (14%)	6 (9%)	34 (52%)
Psychoeducation/consultation (leaflet or phone call on how to cope with various issues: e.g. mental health, sleep, challenging behaviour, parenting groups available, benefit claims)	3 (5%)	6 (9%)	39 (59%)
Counselling	0	0	9 (14%)
Psychotherapy for the child	1 (2%)	1 (2%)	12 (18%)
Cognitive–behavioural therapy for the child	1 (2%)	0	27 (41%)
Video interaction guidance	1 (2%)	0	6 (9%)
Video feedback to promote positive parenting	0	1 (2%)	3 (5%)
Marte Meo	0	0	1 (2%)
Positive parent–child interaction	0	0	0
Paediatric autism communication therapy	0	0	2 (3%)
Play therapy or Theraplay training	0	0	4 (6%)
Lego therapy	0	0	1 (2%)
Sleep therapy	1 (2%)	0	13 (20%)
Triple P	0	0	2 (3%)
Stepping Stones Triple P	0	0	2 (3%)
Incredible Years	1 (2%)	1 (2%)	4 (6%)
Applied behaviour analysis programmes for challenging behaviour (e.g. functional assessment of behaviour and intervention to reduce or replace challenging behaviours)	3 (5%)	0	34 (52%)
Cygnets	0	0	5 (8%)
Hanen programme or other speech and language programme	1 (2%)	1 (2%)	3 (5%)
Non-violent resistance	1 (2%)	2 (3%)	8 (12%)
Other			
Riding the Rapids	1 (2%)	n/a	1 (2%)
PAPAS, PALS, PADDs	2 (3%)	1 (2%)	n/a
Eye movement desensitisation and reprocessing	n/a	n/a	1 (2%)
Dialectical behavioural therapy	n/a	n/a	2 (3%)
Any intervention offered (coded data)	**15 (23%)**	**5 (8%)**	**42 (64%)**

PAPAS, parent awareness programme for autism spectrum; PALS, parent awareness of learning disabilities support group; PADDs, parent awareness of developmental delays.

In terms of support, when respondents were asked to indicate whether their service offers any therapy or psychoeducation, this ranged from 23% to 20% for referral and waiting list, respectively. Free-text data regarding the referral list stage focused mostly on short waiting times as an explanation for why support is not available at this stage (‘short waiting time so intervention not needed’; ‘our service has a very short time from referral to contact (usually 2–6 weeks max) … so no need to offer support while waiting – we would instead prioritise having a short wait time’). One respondent indicated that ‘we are hoping to be able to offer consultations to families in the future and be more available in schools’.

Free-text data regarding the waiting list stage suggested that: some services do periodic check-ins or risk reviews (‘risk review every 12 weeks whilst child on waiting list’), some services signpost to other services (‘psychoeducation and signposting while waiting for treatment’), and some services focus on keeping the waiting time short (‘short wait times of up to 8 weeks’). There was a suggestion that although services may not initiate contact with families, they will respond to family contact during this period: ‘If parents contact us while waiting then we would reply by email/phone as required’.

### Interventions offered to children

During the referral and waiting list phases, psychoeducation was the most frequent support offered, with 5–14% of services offering psychoeducation, depending on the type of psychoeducation and stage of service contact (Table 3). During the active caseload stage, psychoeducation was again the most frequent support, with 52–59% services contacting parents to tell them what their service offers or consulting with them on specific topics such as how to cope with challenging behaviours or sleep problems. Cognitive–behavioural therapy and applied behaviour analytic approaches for behaviour that challenges were the second most frequently reported supports, offered by 41 and 52% of services, respectively. Other named and/or packaged interventions were offered far less frequently. Sleep therapy was offered by 20% of services.

Free-text data provided by respondents under ‘other’ intervention and in response to open-ended questions were used to identify interventions not provided in the list: four additional interventions were reported (Riding the Rapids, dialectical behavioural therapy, eye movement desensitisation and reprocessing, and parent awareness of autism/learning disability/developmental delay support groups). These were offered by very few services (3% or fewer), as can be seen in Table 3.

When considering any intervention offered, 23%, 8% and 64% of specialist mental health services had offered at least one intervention during the referral, waiting list and active caseload stage, respectively, over the previous 12-month period. These results suggest that interventions are least likely to be offered during the waiting list phase, and also that approximately 36% of services did not offer any intervention during the active caseload stage in the previous 12-month period.

## Discussion

The present study captured comprehensive information on service pathways and supports offered by UK specialist mental health services to children with an intellectual disability and suspected mental health problems or behaviours that challenge. Overall, two main findings emerged. While children and families are waiting for therapeutic input, the amount of contact and support they receive from a service varies but appears to be limited; only about 24% (referral stage) to 29% (waiting list stage) of children are contacted once or twice. Considering that median times on referral lists (waiting for assessment) and waiting lists (waiting for treatment) are 29 and 56 days, respectively^6^ (although these are thought to have increased in 2021–22,^7^ the period of the present data), children and their families may be waiting for 1–2 months, and only about one-third of them are contacted during that time.

The second main finding relates to the low levels of therapeutic input across all stages of a child's journey with the service. We looked at data on therapeutic input in two different ways, to capture both general and specific information from service providers. When asked whether their services offer therapy (general question), 23% and 20% of participants indicated that their specialist mental health service provides therapeutic input during the referral and waiting list stages, respectively. When we coded more granular data on types of interventions, we found that 23% (referral stage) and 8% (waiting list stage) of specialist mental health services offered any intervention (Table 3). Taken together, these findings suggest that, at best, only two in ten services offer any therapeutic input at these stages, with input less likely to be offered during the waiting list phase. Although it might not be seen as compatible with a service pathway to offer therapy when a child is on the referral or waiting list, the complexity of presentation needed to meet service referral thresholds is such that a long waiting period with no input may increase complexity and the burden on children and families. The current approach to offering therapeutic input (assessment, formulation, intervention) is likely to be incompatible with an offer of intervention to those at the referral and waiting list stages. However, there are interventions that could help to strengthen families and better prepare them for specialist input: for example, interventions that aim to increase parental self-efficacy, reduce parent stress and/or improve parent–child relationship quality. Future research on intervention effectiveness needs to consider the stage of the service pathway at which an intervention is made available.

More important perhaps was the fact that therapeutic interventions were not uniformly offered during the active caseload phase. Our findings indicated that only 64% of specialist mental health services offered intervention to children on active caseloads, suggesting that 36% of services had not offered any intervention to children on their active caseload in the previous 12 months. In the absence of a named or packaged intervention, services may be offering psychoeducation with a focus on specific needs of the child (59% of services did this in the present study; Table 3). It is not clear why interventions were not available from all services, and our data were not designed to provide a description of all activities performed in specialist mental health services. Services may be signposting to other services or may spend a significant amount of their time and resources diagnosing neurodevelopmental disorders, as opposed to exclusively offering therapeutic support for mental health problems or behaviours that challenge. Children with neurodevelopmental conditions are a population overrepresented in CAMHS services.^8^

Overall, 66 specialist mental health services throughout the UK indicated that between 2021–2022 they offered low levels of contact to referred children with an intellectual disability while these children were waiting for treatment. Psychoeducation, applied behaviour analysis and cognitive–behavioural therapy were the most frequent supports offered, consistent with EU-wide practices^8^ and National Institute for Health and Care Excellence guidelines for mental health and behaviours that challenge in this population.^9,10^ Notably, 36% of respondents did not name a specific packaged intervention on offer during active caseload input over the previous 12 months. Respondents were not a UK-representative sample and, as such, the data may not be an accurate representation of practice across all specialist mental health providers (although there is no UK- or England-wide register of such services^11^). Our study provides only a snapshot of recent activity in specialist mental health services. The findings call for a more standardised pathway to be offered to children and families while they are waiting for therapeutic input, as well as higher levels of offer of interventions to those on the active case list.

## Data Availability

The data that support the findings of this study are available from the corresponding author, V.T., upon reasonable request.

## About the authors

**Vaso Totsika** is Professor of Neurodevelopmental Research in the Division of Psychiatry, University College London, UK; and at the Tavistock & Portman NHS Foundation Trust, London, UK. **Zhixing Yang** is a research assistant in the Division of Psychiatry, University College London, UK. **Lauren Turner** is a research assistant in the Division of Psychiatry, University College London, UK. **Charmaine Kohn** is a trial manager at Tavistock & Portman NHS Foundation Trust, London, UK; and the Division of Psychiatry, University College London, UK. **Angela Hassiotis** is Professor of Psychiatry of Intellectual Disability in the Division of Psychiatry, University College London, UK. **Eilis Kennedy** is a consultant child and adolescent psychiatrist at the Tavistock & Portman NHS Foundation Trust, London, UK. **Michael Absoud** is a consultant in paediatric neurodisability at Evelina London Children's Hospital, South London and Maudsley NHS Foundation Trust, London, UK. **Rachel McNamara** is Director for Brain Health and Mental Wellbeing Trials at the Centre for Trials Research, Cardiff University, UK. **Elizabeth Randell** is a senior trial manager at the Centre for Trials Research, Cardiff University, UK. **Sophie Levitt** is a senior educational psychologist at Brighter Futures for Children, Reading, UK. **Gemma Grant** is Children and Young People Policy lead at the Challenging Behaviour Foundation, Chatham, UK. **Angela Casbard** is Deputy Director of the Cancer Division at the Centre for Trials Research, Cardiff University, UK. **Lauris Jacobs** is a member of the Parent Carer Advisory Group, VIG-LD study, Challenging Behaviour Foundation, Chatham, UK. **Cristina Di Santo** is a member of the Parent Carer Advisory Group, VIG-LD study, Challenging Behaviour Foundation, Chatham, UK. **Claire Buckley** is CAMHS Assistant Clinical Lead at Alder Hey Children's NHS Foundation Trust, Liverpool, UK. **Emma Hignett** is a consultant clinical psychologist, Children's Psychology Professional Lead and Associate Director of Psychology (CYP-LDS) at the Learning Disability Service, Lancashire and South Cumbria NHS Foundation Trust, Preston, UK. **Ashley Liew** is a consultant paediatric neuropsychiatrist at Evelina London Children's Hospital, South London and Maudsley NHS Foundation Trust, London, UK.
